# Supplementary material for: BCR, not TCR, repertoire diversity is associated with favorable COVID-19 prognosis
Source: Front Immunol. 2024 Oct 28;15:1405013. doi: 10.3389/fimmu.2024.1405013 (PMC11550956; doi:10.3389/fimmu.2024.1405013)

Figure S1. Proportion of peripheral blood cell populations. Bar plot of the proportion of cell types shown in Figure 1 for healthy control donors and COVID-19 patients according to disease severity.

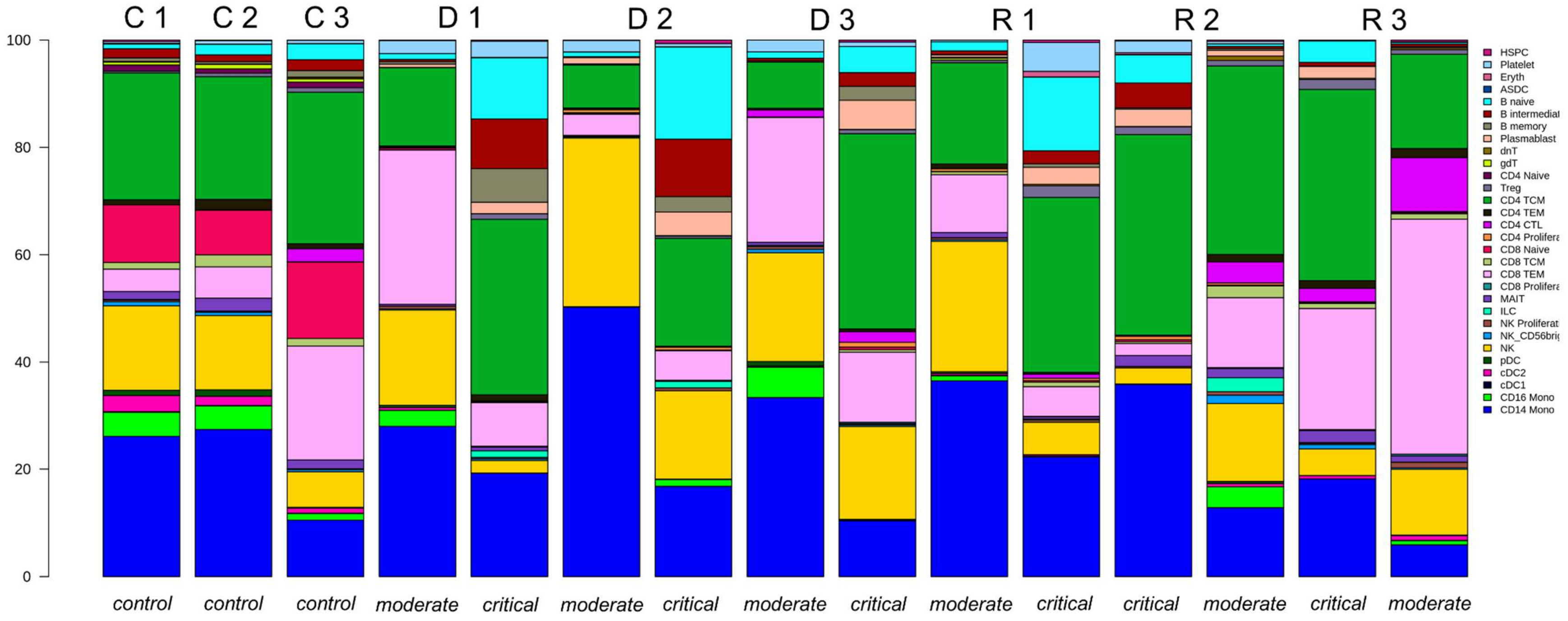

Figure S2. Differentially expressed genes of clonal CD4 and CD8 T cell subsets. For progressing patients (D 1-D 3, and R 1), interferon-stimulating genes and inflammatory genes are upregulated during the critical stage, shown in pink dots. Top ranking genes are annotated. Mitochondrial, ribosomal, and elongation genes are downregulated during the critical stage, in blue dots, which are also the upregulated genes during the moderate stage. For recovering patients (R 2 and R 3), the blue dots are the upregulated genes during the critical state, and the pink dots are the upregulated genes as they recover to the moderate state. Clonal cells are defined as T cell receptors with two or more clones.

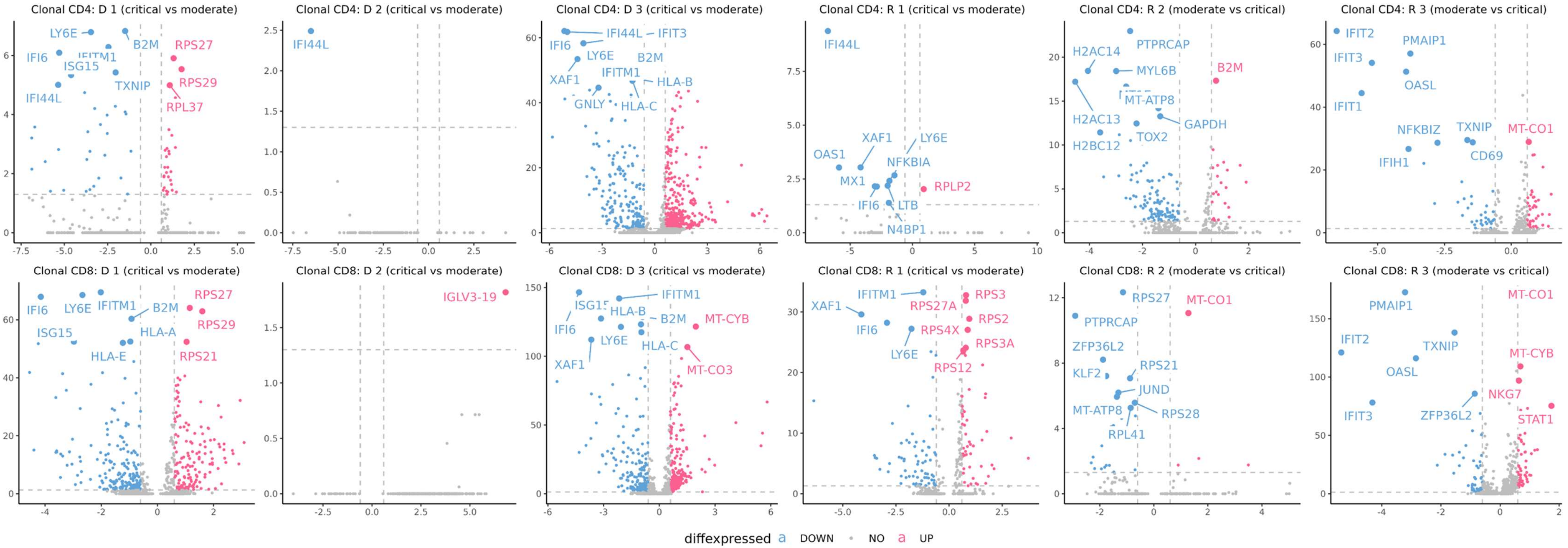

Figure S3. Differentially expressed genes of non-clonal CD4 and CD8 T cell subsets. Non-clonal cells are defined as T cell receptors with unique or unexpanded sequences. There were more gene signatures recovered in non-clonal T cells compared to that in the clonal T cell subsets, also due to the higher frequency of non-clonal T cells. Similar gene signature patterns were observed in non-clonal and clonal T cell subsets.

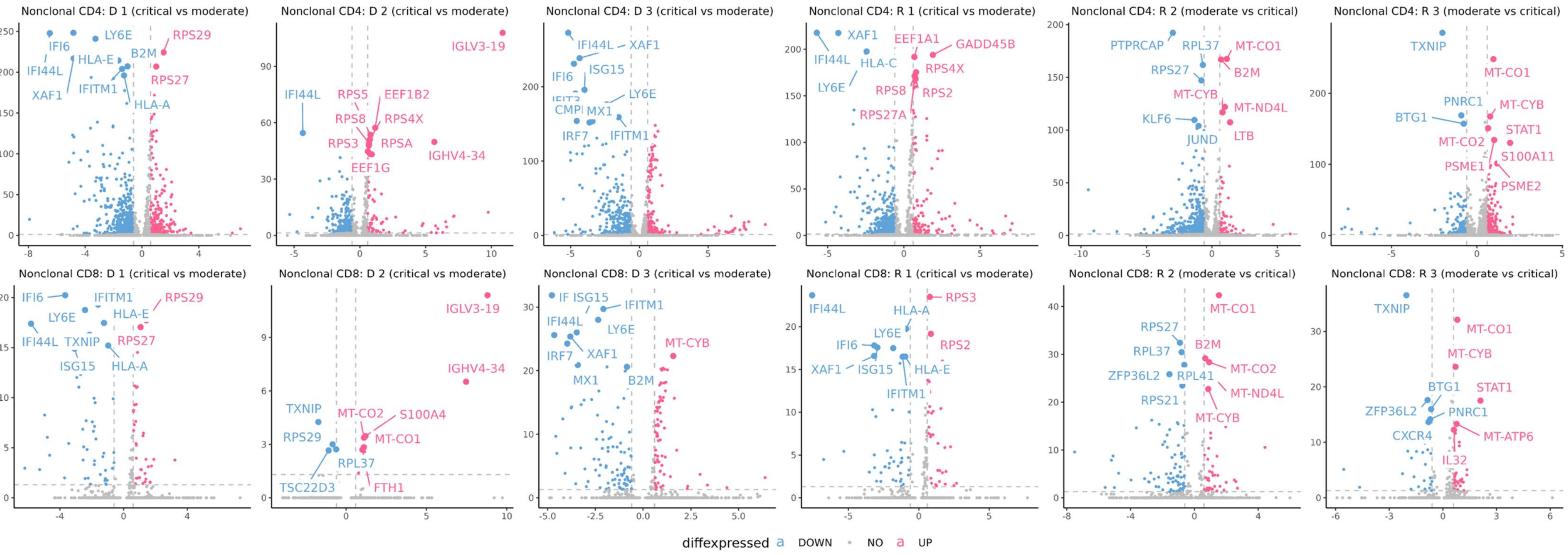

Figure S4. Percentage occupancy of top 50 most abundant clonotypes. Clonotypes with predicted specificity to SARS-COV-2 are shown in red dots. The proportions of the mean occupancy of the Top 50 clonotypes in the whole TCR repertoire and the occupancy of all clonotypes are presented.

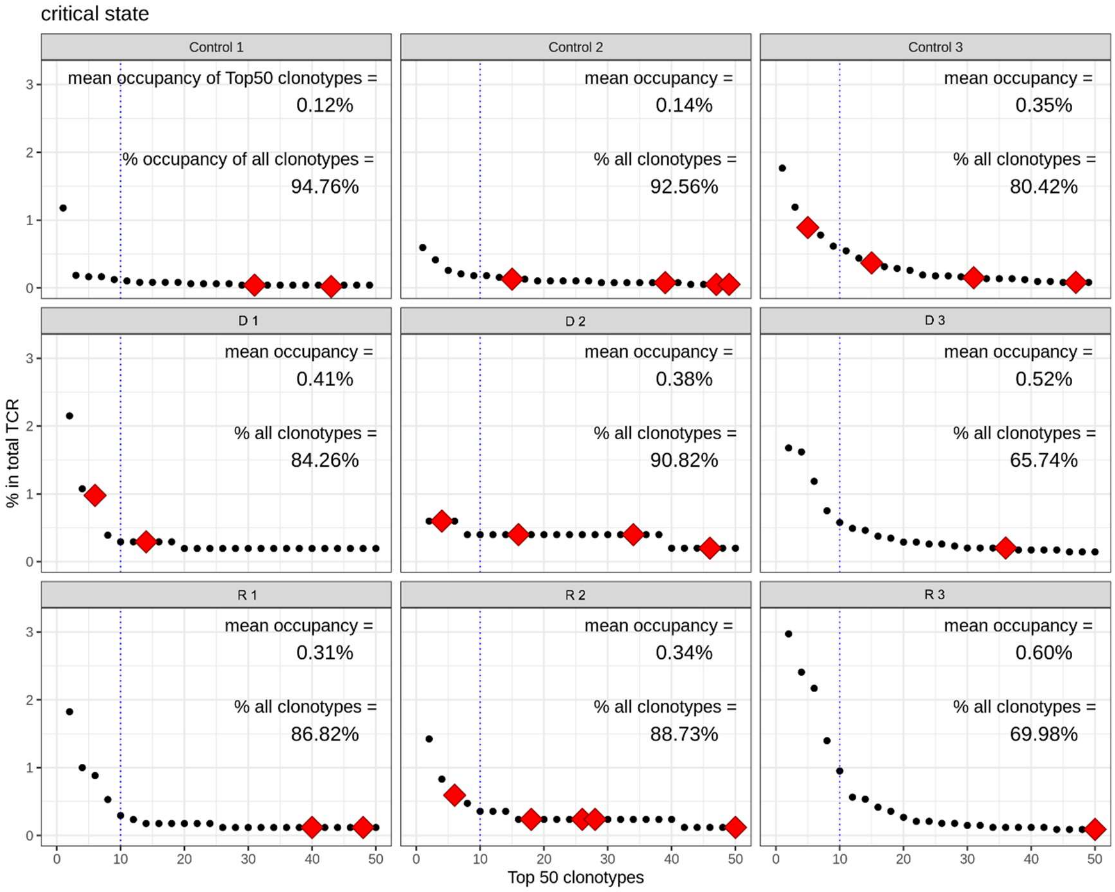

Figure S5. V and J gene usage for T cells. V genes are shown in the x-axis; bar heights represent V gene percentage in each sample; colors represent J genes associated with respective V genes in CD4 TCM **(a)**, and CD8 TEM **(b)**.

a

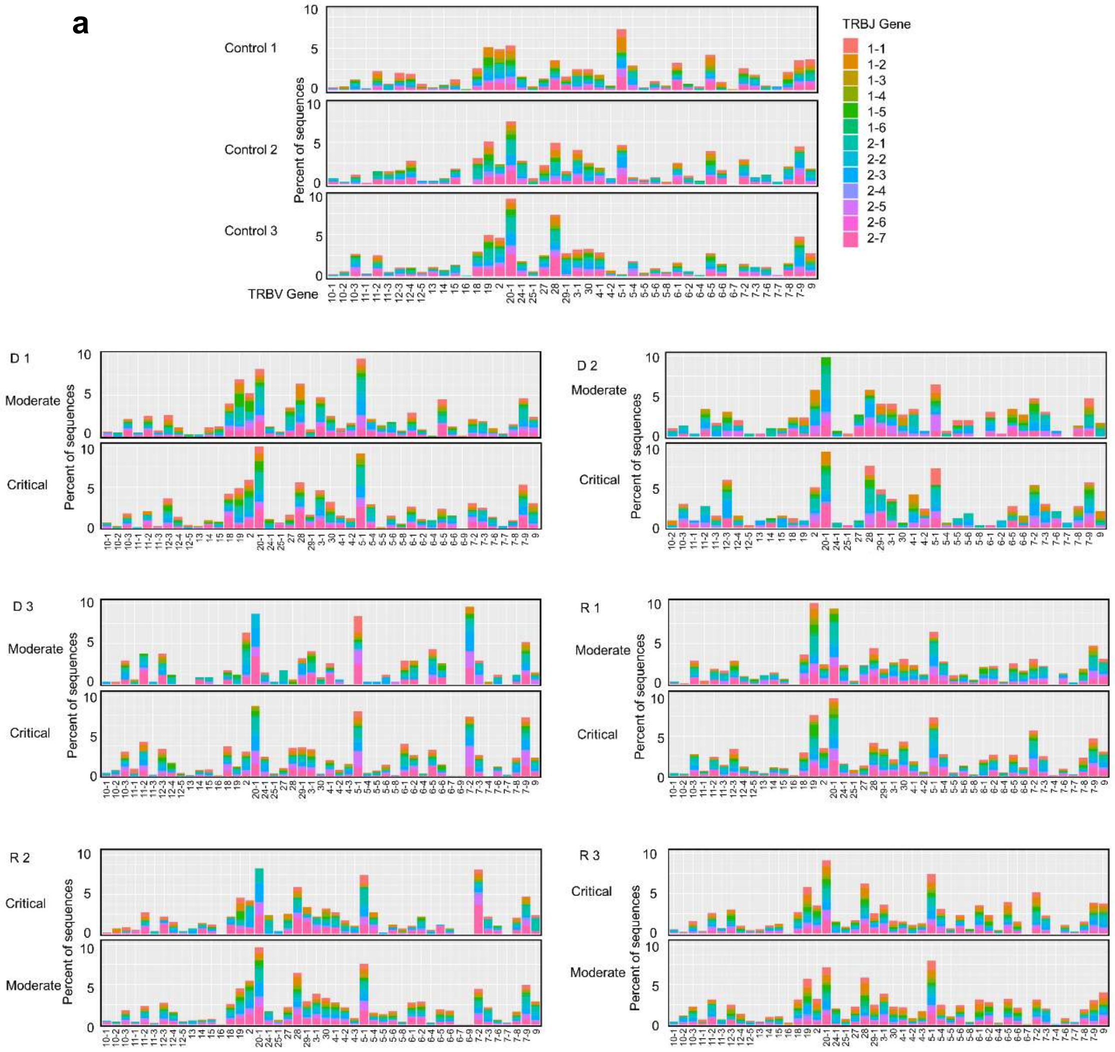

Figure S5. V and J gene usage for T cells (*cont'd*)

b

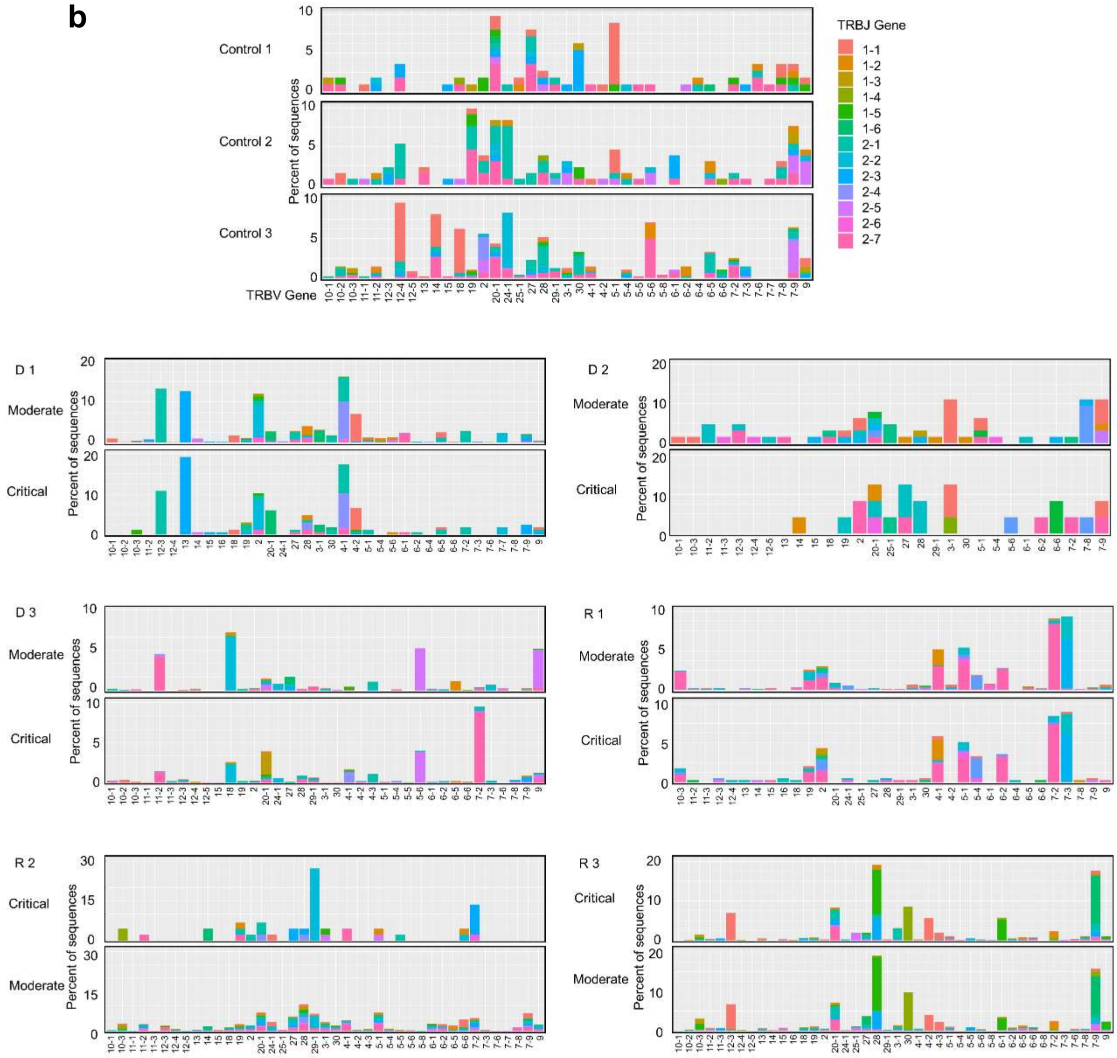

Figure S6. V and J gene usage for B cells. V genes are shown in the x-axis; bar heights represent V gene percentage in each sample; colors represent J genes associated with respective V genes. The scale for the y-axis in COVID-19 samples is 0-20%, except for Pt 2, where, in the critical sample, the IGHV4-34 gene occupies over 40% of the V gene repertoire. Survivors show similar V–J combination proportions to healthy individuals, whereas deceased patients show clonal expansion patterns that are patient-specific, most pronounced in Pt 2’s critical sample.

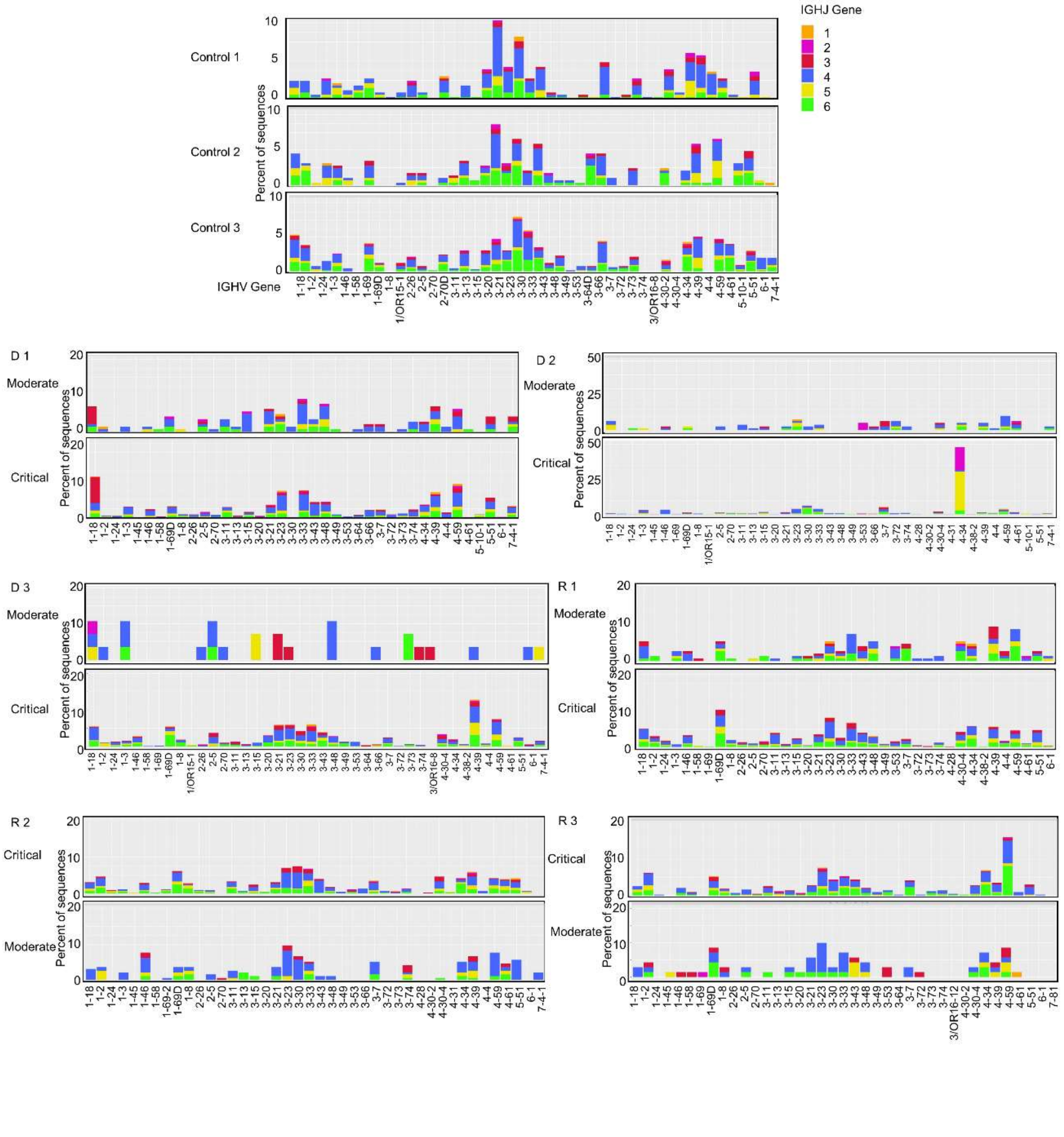

Figure S7. Clonotypes illustrated with somatic hypermutation (SHM) ratio and clonal expansion in relation to IGHV gene usage in critical stage. Each clonotype is represented as bubble with the size indicating the count of each clonotype. The position of each bubble corresponds to the specific HV gene usage and the SHM rate. Arrows in the figure highlight those expanded clonotypes that were selected for subsequent antibody binding assays, indicating their importance in the context of the study.

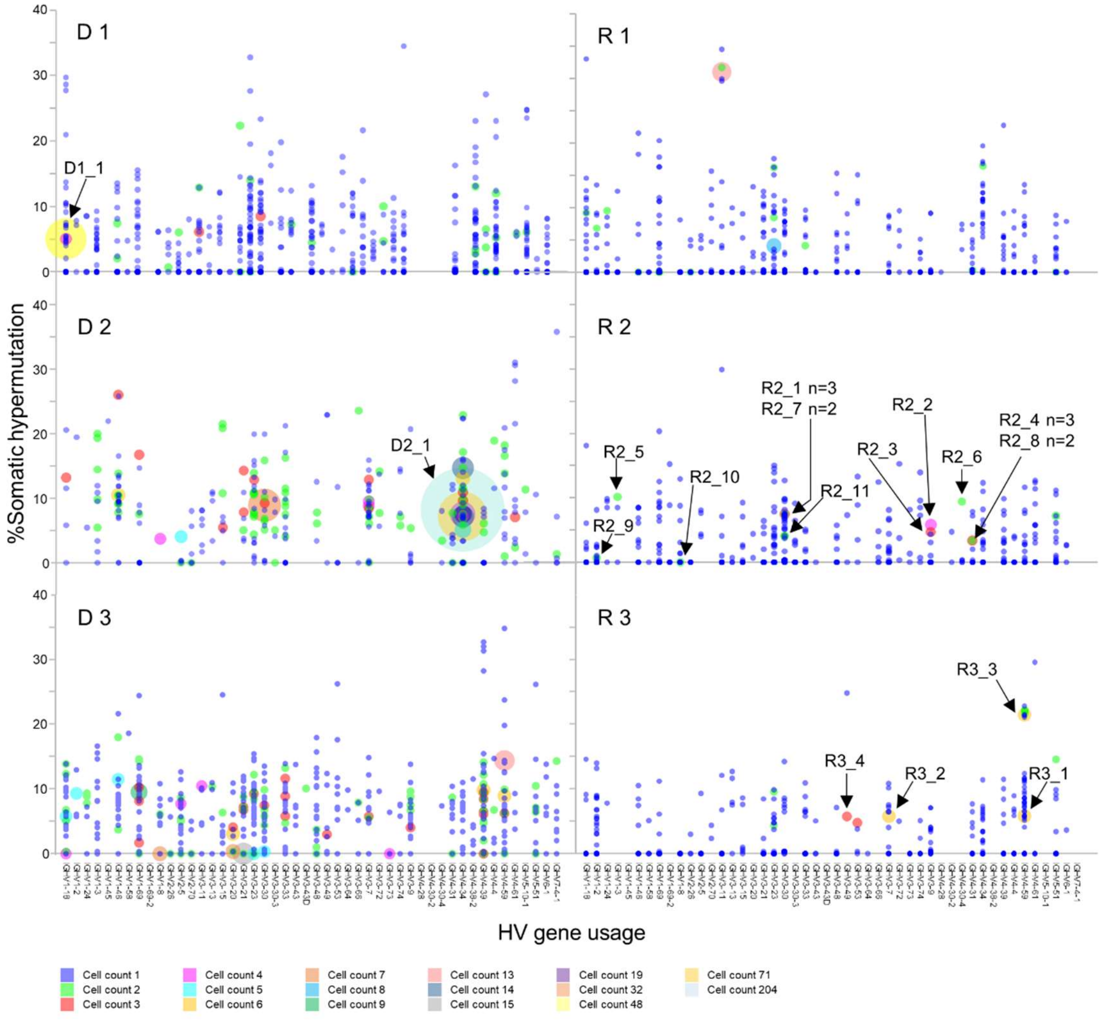

Figure S8. Antibody binding assay against lysate from SARS-CoV-2 infected cells. Recombinant antibodies were generated using sequences from recovered patients (R 2 and R3) and deceased patients (D 1 and D 2). VeroE6/TMPRSS2 and 293T cells were seeded in equal numbers and cultured for 15 hrs. before being infected with SARS-CoV-2 for an additional 6 hrs. Lysates from these infected cells were then immobilized on ELISA plates, followed by incubation with the recombinant antibodies. Antibody binding was detected using HRP-labeled anti-human IgG. Cells not infected with the virus showed greater proliferation, leading to enhanced antibody binding as seen with anti-beta actin antibodies. Notably, higher binding compared to the non-infected samples was observed in p5_3 and in anti-S RBD antibody. These assays were performed in duplicate.

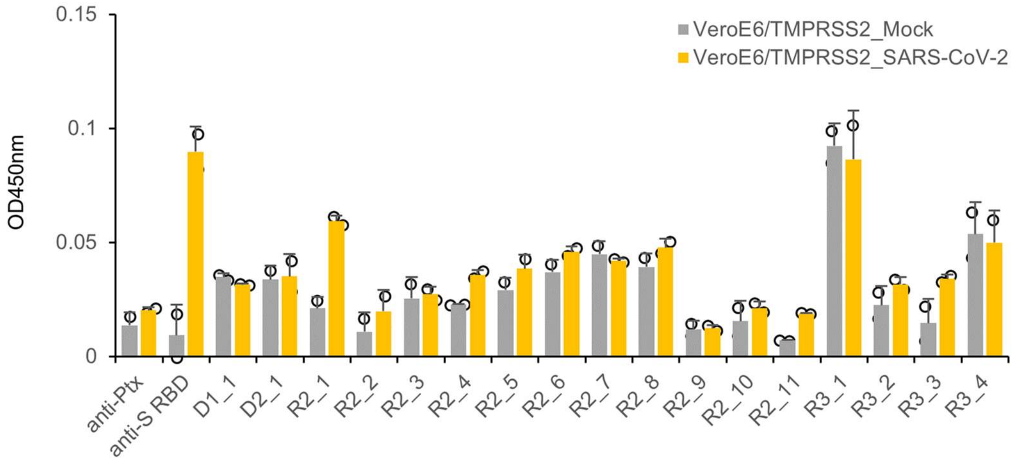

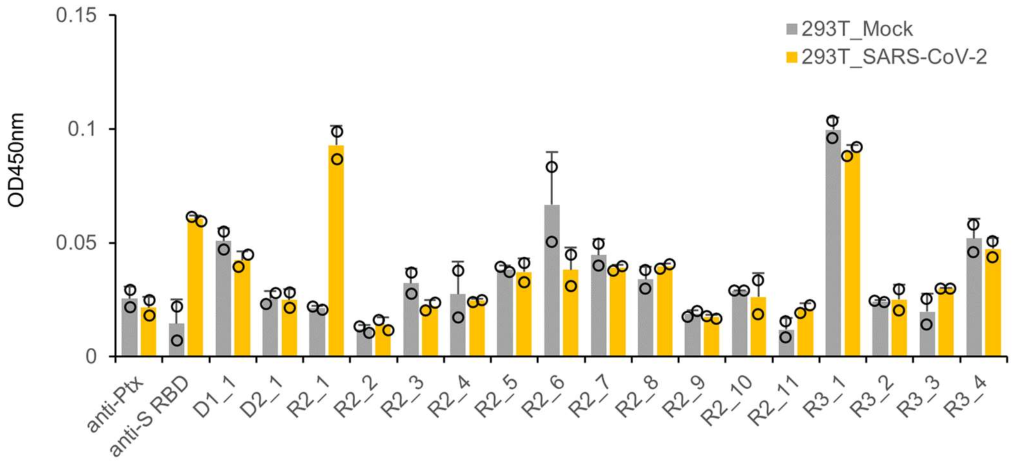

Figure S9. Analysis of Somatic Hypermutations (SHM) in BCR Clonotypes during the Critical Stage of COVID-19. The heavy chain sequences of BCR clone were analyzed the SHM using IMGT/HighV-Quest. We visualized the aggregate number of mutations and the percentage of clonotypes with mutations for each sample using tables and bar graphs. The deceased patients demonstrated more mutations and a greater percentage of mutated clonotypes than others (Welch's t-test, p = 0.041 or 0.016).

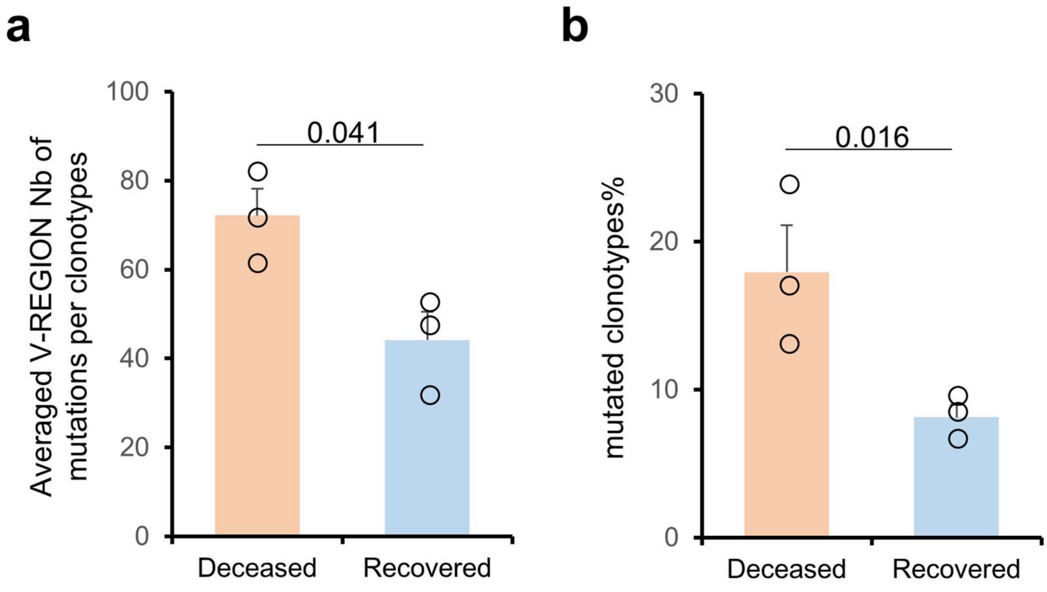

Supplement: Supplementary file 1 [file DataSheet1.pdf]
